# Valproic Acid Induces Cutaneous Wound Healing *In Vivo* and Enhances Keratinocyte Motility

**DOI:** 10.1371/journal.pone.0048791

**Published:** 2012-11-07

**Authors:** Soung-Hoon Lee, Muhammad Zahoor, Jae-Kwan Hwang, Do Sik Min, Kang-Yell Choi

**Affiliations:** 1 Translational Research Center for Protein Function Control, Department of Biotechnology, College of Life Science and Biotechnology, Yonsei University, Seoul, Korea; 2 Department of Molecular Biology, College of Natural Science, Pusan National University, Busan, Korea; University of Florida, United States of America

## Abstract

**Background:**

Cutaneous wound healing is a complex process involving several signaling pathways such as the Wnt and extracellular signal-regulated kinase (ERK) signaling pathways. Valproic acid (VPA) is a commonly used antiepileptic drug that acts on these signaling pathways; however, the effect of VPA on cutaneous wound healing is unknown.

**Methods and Findings:**

We created full-thickness wounds on the backs of C3H mice and then applied VPA. After 7 d, we observed marked healing and reduced wound size in VPA-treated mice. In the neo-epidermis of the wounds, β-catenin and markers for keratinocyte terminal differentiation were increased after VPA treatment. In addition, α-smooth muscle actin (α-SMA), collagen I and collagen III in the wounds were significantly increased. VPA induced proliferation and suppressed apoptosis of cells in the wounds, as determined by Ki67 and terminal deoxynucleotidyl transferase dUTP nick end labeling (TUNEL) staining analyses, respectively. *In vitro*, VPA enhanced the motility of HaCaT keratinocytes by activating Wnt/β-catenin, ERK and phosphatidylinositol 3-kinase (PI3-kinase)/Akt signaling pathways.

**Conclusions:**

VPA enhances cutaneous wound healing in a murine model and induces migration of HaCaT keratinocytes.

## Introduction

Cutaneous wound healing is a complex and dynamic process involving soluble mediators, blood cells, extracellular matrix, and parenchymal cells [1]. The healing process can be divided into three phases: inflammatory, proliferative and remodeling [2], [3]. Signaling pathways crucial for cutaneous wound healing include the Wnt, extracellular signal-regulated kinase (ERK), and phosphatidylinositol 3-kinase (PI3-kinase) signaling pathways [4]–[7].

Wnt signaling pathway plays important roles in development, organogenesis and normal homeostasis [8]–[10]. This pathway is divided into canonical β-catenin-dependent signaling and non-canonical β-catenin-independent signaling pathways [11], both of which are involved in wound healing [5]. In the canonical Wnt signaling pathway, which is often called Wnt/β-catenin pathway, β-catenin is elevated during the proliferative phase of wound healing [12], [13] and regulates mesenchymal cell proliferation, motility, and invasiveness in normal and hyperplastic cutaneous wounds [14]. During the wound healing process, the Wnt/β-catenin signaling pathway also interacts with transforming growth factor (TGF)-β1/Smad signaling [15]–[17]. Furthermore, Wnt3a induced myofibroblast differentiation by up-regulating TGF-β1/Smad signaling pathway [18].

**Figure 1:**
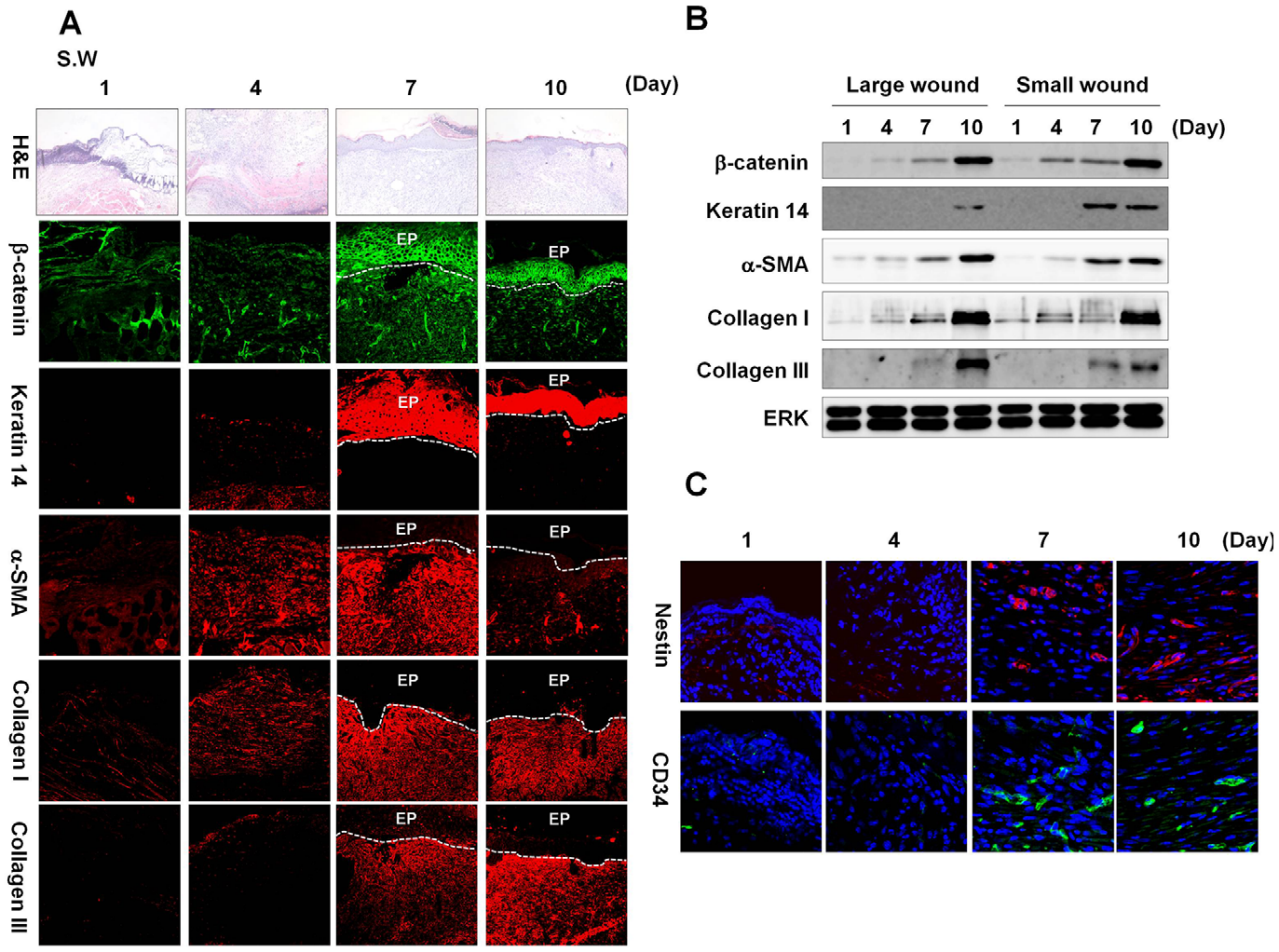
β-catenin status, wound healing and stem cell markers during the healing process in small wounds. Full-thickness wounds (diameter = 0.5 cm) were generated on the backs of 8-week-old C3H mice. Wounded tissues were excised at 1, 4, 7, and 10 d post-wounding, and subjected to H&E staining, immunohistochemical analysis, and western blotting as described in the Materials and Methods. (A) H&E staining (first row panels) (original magnification ×100) and immunohistochemical staining for β-catenin, keratin 14, α-SMA, collagen I, and collagen III (other row panels) in the wounds (original magnification ×200). EP, epidermis. (B) Western blot analyses of β-catenin, keratin 14, α-SMA, collagen I, collagen III, and ERK in large and small wounds. (C) Immunohistochemical analysis of Nestin or CD34 in the wounds at 1, 4, 7, 10 d post-wounding (original magnification ×635).

The ERK and PI3-kinase/Akt signaling pathways are involved in developmental processes, including skin development [19], [20]. In addition, these two pathways are crucial for cutaneous wound healing [6], [7]. Growth factors such as epidermal growth factor (EGF) and basic fibroblast growth factor (bFGF) accelerate keratinocyte migration and epithelialization in skin wound healing by activating the ERK and PI3-kinase/Akt signaling pathways [21], [22]. Conversely, inhibition of these signaling pathways has been shown to impair corneal wound healing [23]. Taken together, these results indicate that the Wnt, ERK and PI3-kinase/Akt signaling pathways may be useful therapeutic targets to enhance wound healing [7], [13], [24].

Valproic acid (VPA; 2-propyl-pentanoic acid) has been prescribed to treat bipolar disorder and epilepsy for several decades [25], [26]. VPA has neuroprotective effects in neurodegenerative diseases including stroke by inhibiting histone deacetylases (HDACs) [27], [28]. VPA also influences several signaling pathways including the Wnt/β-catenin, ERK, and protein kinase C signaling pathways [29]–[31].

In this study, we investigated the effect of VPA on cutaneous wound healing in a murine model. The role of VPA in regulating wound healing was characterized by evaluating its effect on neo-epidermis formation, fibroblast–myofibroblast transition, and cellular proliferation and apoptosis in the wounds. Finally, we assessed the effect of VPA on keratinocyte migration to confirm the role of VPA in wound healing in a human system.

**Figure 2:**
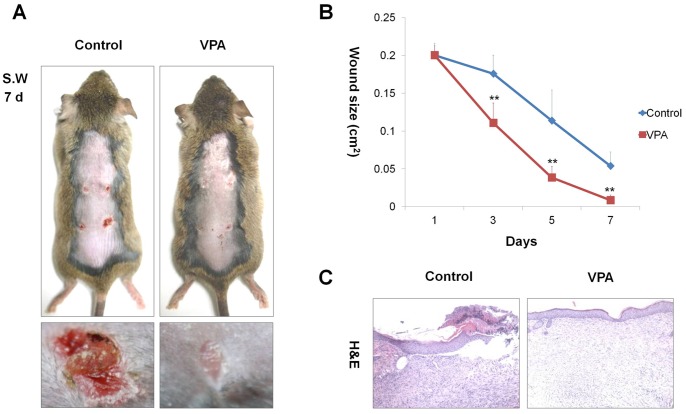
Effects of VPA on cutaneous wound healing. After four full-thickness skin excisions (diameter = 0.5 cm) were made on the backs of 8-week-old C3H mice, 500 mM VPA was topically applied to the wounds daily. Tissues were excised from the wounded area and fixed in paraformaldehyde for H&E staining. (A) Gross images of representative wounds after the 7-d VPA treatment. (B) Relative healing of wounds by VPA treatment. Wound sizes were measured at 1, 3, 5, and 7 d after wounding. Asterisks denote the significant differences between control and test groups as measured by t-test with two asterisks being p<0.005 (n = 10). (C) H&E stained sections of wounded skin treated with or without VPA (original magnification ×100).

## Materials and Methods

### Animals and *in vivo* Wound Healing Assay

Seven-week-old male C3H mice were purchased from Orient Bio Co. and allowed to adapt to their new environment for 1 week. All mouse experiments were conducted in accordance with the Guide for the Care and Use of Laboratory Animals and were approved by the Institutional Review Board of Severance Hospital, Yonsei University College of Medicine (09–013). To determine the effects of VPA on wound healing, 8-week-old C3H mice, whose hair follicles are naturally synchronized in the telogen phase [32] and dermal depths are almost consistent [33], were anesthetized, backs were shaved by hair clipper, cleaned with ethanol, and full-thickness incision wounds were created. VPA (500 mM; Acros) was applied topically to the wounds daily (n = 10). Wound sizes were measured every other day on the assumption that wound depths in each animal are almost constant. The wounded skin tissue was also evaluated by immunohistochemical analysis.

### Cell culture and *in vitro* Wound Healing Assay

HaCaT keratinocytes [34] were cultured in Dulbecco's modified Eagle medium (DMEM, Gibco) containing 10% (v/v) heat-inactivated fetal bovine serum (FBS, Gibco), 100 µg/ml penicillin (Gibco) and 100 µg/ml streptomycin (Gibco), and incubated in 5% (v/v) CO_2_ at 37°C. For the *in vitro* wound healing assay, HaCaT cells (4×10^5^ cells/well) were seeded into 12-well plates in DMEM supplemented with 10% FBS and allowed to adhere overnight. The monolayers were then carefully scratched with sterile pipette tips and incubated with medium containing 2% serum with or without 100 µM VPA. After 24 h, the cells were washed once with cold phosphate buffered saline (PBS), fixed in 4% paraformaldehyde for 15 min at room temperature, and stained with 2% (w/v) crystal violet. The wound closure rate was measured using NIS-Elements imaging software (Nikon) (n = 3).

**Figure 3:**
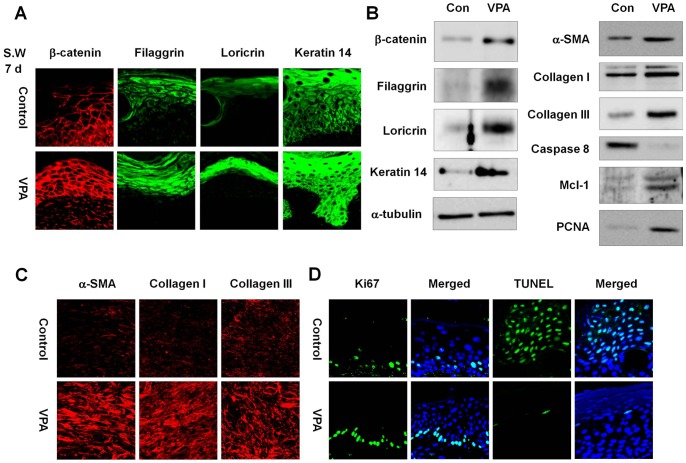
Effects of VPA on levels of β-catenin, wound healing, proliferation, and apoptosis markers in small wounds. The wounded skin of 8-week-old male C3H mice was treated daily with 500 mM VPA for 7 days. A portion of the wounded tissues was excised and frozen in liquid nitrogen for Western blot analysis, and the remaining tissue was fixed in paraformaldehyde. (A) Immunohistochemical analysis of β-catenin, filaggrin, loricrin, or keratin 14 in the neo-epidermis of control and VPA-treated wounds (original magnification ×635). (B) Western blot analysis of β-catenin, filaggrin, loricrin, keratin 14, α-tubulin, α-SMA, collagen I, collagen III, caspase 8, Mcl-1, or PCNA in the control and VPA-treated wounds. (C) Immunohistochemical analysis of α-SMA collagen I, or collagen III in the control and VPA-treated wounds (original magnification ×635). (D) The control and VPA-treated wounds were stained for Ki67 and evaluated using the TUNEL assay. The nuclei were stained with DAPI (original magnification ×635).

### siRNA Preparation and Transfection

The human β-catenin small interfering RNA (siRNA) target sequences were 5′-GAAACGGCTTTCAGTTGAG-3′ and 5′-AAACTACTGTGGACCACAAGC-3′. The siRNAs were transfected into HaCaT cells using Lipofectamine Plus reagent (Invitrogen) at a final concentration of 100 nM. Transfected cells were grown for 24 h in 5% (v/v) CO_2_ at 37°C. The cells were scratched and incubated for 24 h with medium containing 2% serum with or without 100 µM VPA.

### Western Blot Analysis

Cells and ground tissue powder were lysed with RIPA buffer (150 mM NaCl, 10 mM Tris, pH 7.2, 0.1% sodium dodecyl sulfate (SDS), 1.0% Triton X-100, 1% sodium deoxycholate, 5 mM EDTA). Proteins were separated by sodium dodecyl sulfate-polyacrylamide gel electrophoresis (SDS-PAGE) and transferred onto PROTRAN® nitrocellulose membranes (Schleicher and Schuell Co.). After blocking with PBS containing 5% nonfat dry skim milk and 0.07% (v/v) Tween 20, the membranes were incubated with primary antibodies against β-catenin (Santa Cruz Biotechnology; 1∶1000), fillagrin (Covance; 1∶1000), loricrin (Covance; 1∶1000), keratin 14 (Covance; 1∶1000), α-tubulin (Oncogene Research Products; 1∶5000), α-SMA (Abcam; 1∶1000), collagen I (Abcam; 1∶1000), collagen III (Abcam; 1∶5000), caspase 8 (Cell Signaling Technology; 1∶500), myeloid cell leukemia sequence 1 (Mcl-1, Cell Signaling Technology; 1∶500), proliferating cell nuclear antigen (PCNA, Santa Cruz Biotechnology; 1∶500), E-cadherin (Cell Signaling Technology; 1∶1000), p-ERK (Santa Cruz Biotechnology; 1∶500), or p-Akt (Santa Cruz Biotechnology; 1∶1000) overnight at 4°C. Secondary antibodies were horseradish peroxidase-conjugated anti-mouse (Cell Signaling Technology) or anti-rabbit (Bio-Rad) antibodies. Proteins were visualized by enhanced chemiluminescence (Amersham Bioscienc) using a luminescent image analyzer, LAS-3000 (Fujifilm).

**Figure 4:**
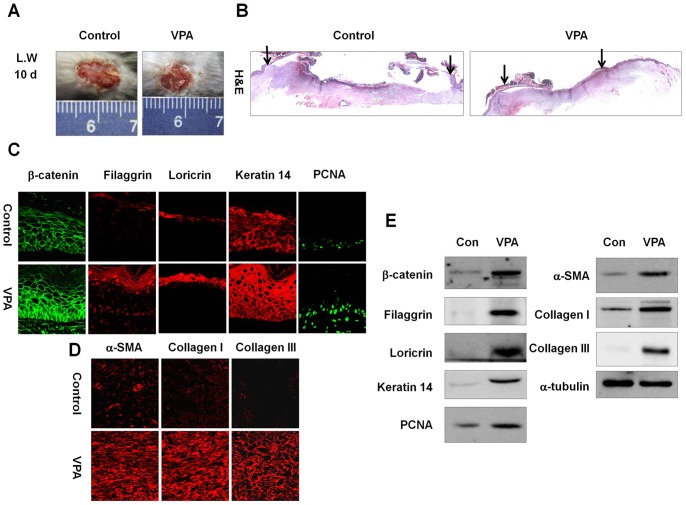
Effects of VPA on cutaneous wound healing in large wounds. A full-thickness skin excision (diameter = 1.5 cm) was made on the backs of 8-week-old C3H mice, and 500 mM VPA was topically applied to the wounds daily. Tissues were excised from the wounded area and fixed in paraformaldehyde for immunohistochemistry or frozen in liquid nitrogen for Western blotting. (A) Representative gross images of wounded skin after 10-d VPA treatment. (B) Representative H&E stained tissues of wounded skin treated with or without VPA. Arrows represent the wound edge (original magnification ×40). (C) Immunohistochemical analysis of β-catenin, filaggrin, loricrin, keratin 14, or PCNA in the neo-epidermis of control and VPA-treated wounds (original magnification ×635). (D) Immunohistochemical analysis of α-SMA, collagen I, or collagen III in the control and VPA-treated wounds (original magnification ×635). (E) Western blot analysis of β-catenin, filaggrin, loricrin, keratin 14, PCNA, α-SMA, collagen I, collagen III, or α-tubulin in the control and VPA-treated wounds.

### Hematoxylin and Eosin Staining

Tissues were embedded in paraffin and cut into 4-µm sections. The sections were deparaffinized in three changes of xylene and rehydrated through a graded ethanol series. The sections were stained with hematoxylin for 5 min and with eosin for 1 min. The slides were then dehydrated through a graded alcohol series, cleared in xylene and mounted in Permount (Fisher Scientific). The hematoxylin and eosin (H&E)-stained slides were visualized using a bright-field optical microscope (Nikon TE-200U).

### Immunohistochemistry

Paraformaldehyde-fixed paraffin-embedded tissues were cut into 4-µm sections. The slides were deparaffinized in xylene and rehydrated through a graded alcohol series. For antigen retrieval, the slides were autoclaved in 10 mM sodium citrate buffer. Sections were pre-incubated in PBS and then blocked in PBS containing 5% bovine serum albumin (BSA) and 1% goat serum for 30 min at room temperature. The sections were incubated overnight at 4°C with primary antibodies against β-catenin (BD Transduction Laboratory; 1∶100), filaggrin (Covance; 1∶500), loricrin (Covance; 1∶500), keratin 14 (Covance; 1∶500), Ki67 (Abcam; 1∶500), α-SMA (Abcam; 1∶100), collagen I (Abcam; 1∶100), collagen III (Abcam; 1∶200), and or PCNA (Santa Cruz; 1∶500). The sections were rinsed with PBS and incubated with IgG secondary antibody conjugated to Alexa Fluor 488 or Alexa Fluor 555 (Molecular Probes; 1∶400) for 1 h at room temperature, and counterstained with 4'-6-diamidino-2-phenylindole (DAPI, Boehringer Mannheim; 1∶5000). Fluorescent signals were visualized on a LSM510 META confocal microscope (Carl Zeiss) and quantified using TissueQuest image analysis software (TissueGnostics GmbH) (n = 5). The mean relative intensity was calculated by integrating fluorescence intensity around each individual nucleus.

### Apoptosis Assay

Tissue sections (4-µm thick) were deparaffinized and rehydrated. The slides were incubated in 20 µg/ml proteinase K for 15 min at room temperature, and washed twice with PBS for 5 min. Terminal deoxynucleotidyl transferase dUTP nick end labeling (TUNEL) staining was performed using the ApopTag Fluorescein *In Situ* Apoptosis Detection Kit (Chemicon).

### Immunocytochemistry

Cells were fixed with 4% paraformaldehyde for 15 min at room temperature and washed with PBS. For permeabilization, cells were treated with 0.1% Triton X-100 for 15 min at room temperature. After blocking with 5% BSA and 1% goat serum in PBS for 30 min at room temperature, the cells were incubated with mouse anti-β-catenin antibody (BD Transduction Laboratories; 1∶100) or rabbit E-cadherin antibody (Cell Signaling Technology; 1∶100) overnight at 4°C. The cells were rinsed with PBS and then incubated with Alexa Fluor 488-conjugated goat anti-mouse antibody or anti-rabbit antibody (Molecular Probes; 1∶400) for 1 h at room temperature, counterstained with DAPI (Boehringer Mannheim; 1∶5000), and examined under confocal microscope, LSM510 META (Carl Zeiss).

**Figure 5:**
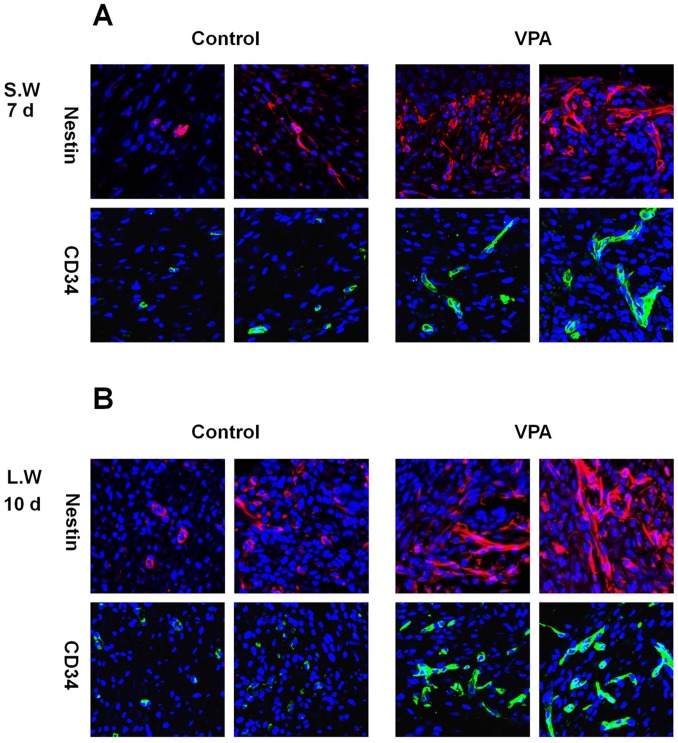
Effects of VPA on the expression of stem cell markers in wounds. A full-thickness skin excision (diameter = 0.5 cm or 1.5 cm) was made on the backs of 8-week-old C3H mice, and 500 mM VPA was topically applied to the wounds daily. (A) Immunohistochemical analysis of Nestin or CD34 in the control and VPA-treated small wounds (original magnification ×635). (B) Immunohistochemical analysis of Nestin or CD34 in the control and VPA-treated large wounds (original magnification ×635).

### Statistical Analysis

Statistical analyses were performed using unpaired two-tailed Student's t-test. Asterisks indicate the statistically significant differences with one asterisk being p<0.05 and two asterisks being p<0.005.

## Results

### The Wnt/β-catenin Signaling Pathway is Activated during the Wound Healing Process

We investigated the activation status of the Wnt/β-catenin signaling pathway during the wound healing process in a murine model to characterize the role of this pathway in cutaneous wound healing. We created full-thickness wounds (diameter = 0.5 cm) on the backs of mice and monitored levels of β-catenin and wound healing markers. β-Catenin was highly expressed in epidermal keratinocytes and dermal fibroblasts at 7 and 10 d after wounding (Figure 1A). The expression of keratin 14, a marker of undifferentiated keratinocytes, was also high in epidermis at 7 and 10 d post-wounding (Figure 1A). The α-smooth muscle actin (α-SMA) was gradually increased in dermal fibroblasts and its level was maximal at 7 d post-wounding (Figure 1A). Expression levels of collagen I and collagen III were also gradually increased in dermal fibroblasts of cutaneous wounds (Figure 1A). In large cutaneous wounds (diameter = 1.5 cm), β-catenin and wound healing markers were also progressively increased during the wound healing process of mice (Figure S1A). The high expressions of β-catenin and wound healing markers, especially at 7 and 10 d post-wounding, were also confirmed by western blot analyses (Figure 1B). We founded that adult stem cell markers, Nestin and CD34 were expressed at 7 and 10 d after wounding (Figures 1C and S1B).

### VPA Promotes Cutaneous Wound Healing

Because of the relationship between Wnt/β-catenin signaling pathway and wound healing [4], [5], we evaluated the effectiveness of VPA on cutaneous wound healing. With daily application of VPA to the wound area (diameter = 0.5 cm) of C3H mice, the wounds were markedly reduced in size and almost completely re-epithelialized after 7 d (Figures 2A and 2B). Histological analysis also showed restitution of normal tissue structure following application of VPA (Figure 2C).

**Figure 6:**
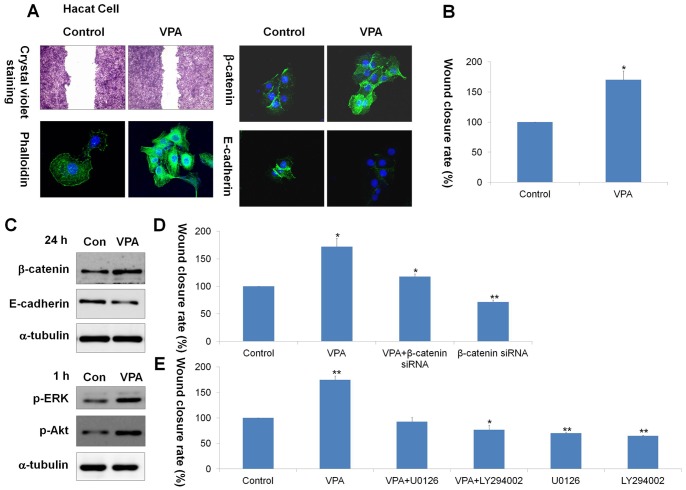
Effects of VPA, β-catenin siRNA, U0126, or LY294002 on HaCaT keratinocyte migration. HaCaT keratinocytes were used to determine the effect of VPA on human keratinocyte migration. Cells were maintained in DMEM supplemented with 10% heat-inactivated FBS, streptomycin (100 µg/ml), and penicillin (100 µg/ml) in 5% CO_2_ at 37°C. After scratch wounding with sterile pipette tips, HaCaT keratinocytes were incubated with medium containing 2% serum with or without 100 µM VPA for 24 h. (A) Cells treated with or without 100 µM VPA were stained with crystal violet for 24 h (first row panel; original magnification ×40). Immunocytochemical analyses of phalloidin, β-catenin, or E-cadherin in control and VPA treated HaCaT cells (second, third, and fourth row panel; original magnification ×400). (B) The relative wound closure rate of HaCat cells treated with or without 100 µM VPA. The wound closure rate was measured using NIS-Elements imaging software. Asterisks denote the significant differences between control and test groups as measured by t-test with one asterisk being p<0.05 (n = 3). (C) Western blot analysis of β-catenin, E-cadherin, p-ERK, p-Akt, or α-tubulin in control and VPA-treated HaCaT cells. (D) HaCaT cells were transfected with 100 nM β-catenin siRNA before VPA treatment. The wound closure rate was measured using NIS-Elements imaging software. Asterisks indicate the statistically significant differences as measured by t-test with one asterisk being p<0.05 and two asterisks being p<0.005 (n = 3). (E) 10 µM U0126 or LY294002 was pre-treated for 1 h before VPA treatment, and the wound closure rate was measured after 24-h VPA treatment. One asterisk means p<0.05 and asterisks being p<0.005 (n = 3).

### VPA Increases β-catenin, Keratin 14, and Markers of Keratinocyte Terminal Differentiation in the Wound Neo-epidermis

VPA is known to activate the Wnt/β-catenin signaling pathway by inhibiting GSK3β [35], [36]. To determine the effect of VPA on this pathway in our mouse model of wound healing, β-catenin expression was evaluated in VPA-treated wounds. We observed increased β-catenin expression in the wound neo-epidermis after a 7-d application of VPA (Figures 3A and S2A). Both filaggrin and loricrin, markers of keratinocyte terminal differentiation involved in neo-epidermis formation, and keratin 14 were induced by VPA (Figures 3A and S2A). Western blot analysis confirmed increased levels of β-catenin, filaggrin, loricrin, and keratin 14 in wounds treated with VPA (Figures 3B and S3A).

### VPA Induces Fibroblast-to-myofibroblast Transition

Fibroblast-to-myofibroblast transition is an important step in cutaneous wound healing [37]. Protein levels of α-SMA, collagen I, and collagen III, markers of myofibroblast differentiation, were significantly increased by treatment of VPA onto the wounds, as shown by Western blot and immunohistochemical analysis (Figures 3B, 3C, S2B and S3B).

### VPA Induces Cell Proliferation and Inhibits Apoptosis in the Wound

The increase in cell proliferation and decrease in apoptosis are important phenomena in early wound healing [38]. We stained cells for Ki67 to detect cell proliferation and performed the terminal deoxynucleotidyl transferase dUTP nick end labeling (TUNEL) staining to evaluate apoptosis in VPA-treated wounds. Our results showed that VPA stimulated cell proliferation and suppressed apoptosis in the wound (Figure 3D). Moreover, Ki67 was expressed in a linear pattern in the epidermal layer of VPA-treated wounds, but was expressed in a scattered pattern in the vehicle-treated wounds (Figure 3D). Caspase 8, mediator of apoptosis during wound healing [39], was significantly decreased by application of VPA onto the wounds (Figure 3B). In contrast, protein levels of Mcl-1, an anti-apoptotic protein, and PCNA, a proliferation marker, were increased in VPA-treated wounds (Figure 3B).

### VPA enhances cutaneous healing in large wounds

To investigative the effect of VPA on cutaneous wounds of different sizes, we created large cutaneous wounds (diameter = 1.5 cm) on the dorsal skin of C3H mice and topically applied VPA to the wound. VPA accelerated healing of large cutaneous wounds, as determined by measurements of wound diameters (Figure S4), and the wound size was markedly reduced in VPA-treated wounds after 10 d (Figure 4A). Histological analysis also showed that the distance between the wound edges were decreased by VPA (Figure 4B). Levels of β-catenin, filaggrin, loricrin, keratin 14, and PCNA in the neo-epidermis of VPA-treated wounds were significantly increased (Figure 4C). In addition, α-SMA, collagen I, and collagen III were significantly increased by VPA (Figure 4D). Western blot analyses also confirmed that β-catenin, filaggrin, loricrin, keratin 14, α-SMA, and collagen I were increased after VPA treatment (Figure 4E).

### VPA Induces the Expression of Stem Cell Markers in the Wound

Multipotent adult stem cells contribute to cutaneous wound healing [40]–[42]. To investigative the effect of VPA on stem cells in the wounds, the expression levels of stem cell markers, Nestin and CD34 were determined by immunohistochemical anlaysis. VPA induced the expression of Nestin and CD34 in dermis of small wounds after 7 d (Figures 5A and S5A). Nestin- or CD34-expressing cells were also increased in large wounds after a 10-d application of VPA (Figures 5B and S5B).

### VPA Enhances Motility of HaCaT Keratinocytes by Activating Wnt/β-catenin, ERK and PI3-kinase/Akt Signaling Pathways

To determine whether VPA can influence keratinocyte migration in a human system, we evaluated the effect of VPA on HaCaT keratinocyte migration after scratch wounding. After 24 h, VPA enhanced narrowing of the scratch wound in HaCaT keratinocytes (Figures 6A and 6B), showing a maximum enhancement at the concentration of 100 µM (Figure S6). VPA-treated cells exhibited more stress fibers and a thicker cortical network than control cells, as demonstrated by phalloidin staining (Figure 6A). β-Catenin level was increased by 24-h VPA treatement, but E-cadherin level was decreased (Figures 6A and 6C). The effect of Wnt/β-catenin signaling pathway on VPA-induced keratinocyte migration was analyzed by using β-catenin siRNA transfection. VPA increased nuclear translocation of β-catenin, but this VPA effect was significantly reduced by β-catenin knockdown, as shown by immunocytochemical and western blot analyses (Figures S7A and S7C). The VPA-induced keratinocyte migration was blocked (Figures 6D and S8) and decrease of E-cadherin level by VPA treatment was recovered by β-catenin siRNA (Figures S7B and S7C).

The ERK and PI3-kinase/Akt signaling pathways are known to be involved in keratinocyte migration [21], [22]. Therefore, we next investigated the involvement of ERK and PI3-kinase/Akt signaling pathways in VPA-induced keratinocyte migration. Western and immunocytochemical analysis showed that the activities of ERK and Akt were significantly increased by 1-h VPA treatment (Figures 6C and S9). Such activations were specifically abolished by pre-treatment with U0126 or LY294002, selective inhibitors for MEK and PI3- kinase, respectively (Figure S9). After 24 h, the VPA-induced keratinocyte migration was also blocked by U0126 or LY294002 (Figures 6E and S10).

## Discussion

VPA is an antiseizure drug commonly prescribed for epilepsy and used as a mood stabilizer for bipolar disorder over the last several decades. Previous studies have shown that VPA exerts differential effect on cell proliferation and migration. For example, VPA suppresses breast cancer cell migration by specifically targeting HDAC2 and down-regulating survivin [43]. VPA also inhibits proliferation and migration of prostate cancer cells by hyperacetylation of histone H3 and H4 [44]. However, VPA enhances mesenchymal stem cell migration by inhibiting GSK3β and increasing protein levels of matrix metalloproteinase-9 (MMP-9) [45]. VPA also stimulates the proliferation of hematopoietic stem cells by inhibiting GSK3β and up-regulating HOXB4, a target of Wnt/β-catenin signaling pathway [46]. Thus, VPA regulates proliferation and migration in a cell-specific or tissue-specific manner. In this study, we found that VPA induced proliferation and suppressed apoptosis in wounds and enhanced motility of HaCaT keratinocytes. The finding that VPA increases keratinocyte proliferation is consistent with the results of our previous studies reporting that VPA induces hair regeneration [47].

VPA has been shown to activate Wnt/β-catenin signaling pathway by inhibiting GSK3β [35], [36]. We found that VPA increased markers of keratinocyte terminal differentiation, which are regulated by Wnt/β-catenin signaling pathway [48], [49] in the wound neo-epidermis. Wnt/β-catenin signaling pathway is also important for regulation of cell proliferation and motility in the wounds [13], [14]. Although hypertrophic scars and keloids are the result of dysregulated Wnt/β-catenin signaling pathway [50], we believed that the moderate regulation of Wnt/β-catenin signaling pathway in the normal cutaneous wounds or other types of wounds is important for enhancing wound healing.

During early wound healing, cell proliferation is controlled by a decrease in apoptosis [13]. VPA has been shown to activate the ERK and PI3-kinase/Akt signaling pathways [51], [52]. Our results indicate that VPA may regulate proliferation in early wound healing via the ERK and PI3-kinase/Akt signaling pathways.

We found that VPA induced the expression of stem cell markers, Nestin and CD34. Nestin-expressing interfollicular blood vessel network contributes to skin wound healing [41]. It is also known that CD34 positive cell transplantation improves wound healing by increasing neo-vascularization [42]. The induction of Nestin and CD34 by VPA may be important in the enhancement of efficacy of stem cell therapy in cutaneous wounds.

Taken together, our results suggest that VPA induces cutaneous wound healing in murine model and enhances HaCaT keratinocyte migration. VPA induced complete re-epithelialization in wounds of mice at an earlier time than other agents, although the direct comparison of the effectiveness of the healing between VPA and those agents are difficult [53], [54]. In terms of safety, drugs used for cuatenous wound healing such as growth factors and small molecules often result in potential side effects [55]. However, VPA, which has been used for several decades to safely treat neurological disorders [25], can potentially be applied without significant side effects. Therefore, VPA may be useful for the development of therapies to enhance wound healing.

## Supporting Information

Figure S1 β-catenin status, wound healing and stem cell markers during the healing process in large wounds. Full-thickness wounds (diameter = 1.5 cm) were generated on the backs of 8-week-old C3H mice. Wounded tissues were excised from CH3 mice at 1, 4, 7, and 10 d post-wounding, and subjected to H&E staining and immunohistochemical analyses. (A) H&E staining (first row panels) (original magnification ×100) and immunohistochemical staining for β-catenin, keratin 14, α-SMA, collagen I, and collagen III (other row panels) in the wounds (original magnification ×200). EP, epidermis. (B) Immunohistochemical analysis of Nestin or CD34 in the wounds at 1, 4, 7, and 10 d post-wounding (original magnification ×635).
(TIF)

Figure S2 Effects of VPA on levels of β-catenin, wound healing markers in small wounds. The wounded skin of 8-week-old male C3H mice was treated daily with 500 mM VPA for 7 days. Wounded skin was fixed in paraformaldehyde overnight. (A) Immunohistochemistry was performed with β-catenin, filaggrin, loricrin, or keratin 14 antibodies in the neo-epidermis of control and VPA-treated wounds. The protein levels were quantified using TissueQuest analysis software. Asterisks denote the significant differences between control and test groups as measured by t-test with one asterisk being p<0.05, two asterisks being p<0.005 (n = 5).
(TIF)

Figure S3 Effects of VPA on levels of β-catenin, wound healing markers in small wounds. The relative protein expression was calculated as the ratio of each protein level to α-tubulin level. The software used for the quantification was Multi-Gauge V 3.0 (Fujifilm). Asterisks denote the significant differences between control and test groups as measured by t-test with two asterisks being p<0.005 (n = 5).
(TIF)

Figure S4 Quantitative data for effects of VPA on cutaneous wound healing in large wounds. A full-thickness skin excision (diameter = 1.5 cm) was made on the backs of 8-week-old C3H mice, and 500 mM VPA was topically applied to the wounds daily (Figure 4A). Wound sizes were measured at 1, 3, 5, 7, and 9 d after wounding. Asterisks denote the significant differences between control and test groups as measured by t-test with two asterisks being p<0.005 (n = 10).
(TIF)

Figure S5 Effects of VPA on the expression of stem cell markers in wounds (Low magnification images). A full-thickness skin excision (diameter = 0.5 cm or 1.5 cm) was made on the backs of 8-week-old C3H mice, and 500 mM VPA was topically applied to the wounds daily. (A) Lowly magnified image of Fig. 5A (original magnification ×200). (B) Lowly magnified image of Fig. 5B (original magnification ×200).
(TIF)

Figure S6 Effects of VPA concentration on HaCaT HaCaT keratinocyte migration. Hacat cells were treated with different concentrations of VPA for 24 hours. (A) Migrating cells were stained with crystal violet (original magnification ×40). (B) The relative wound closure rate was measured using NIS-Elements imaging software. Asterisks denote the significant differences between control and test groups as measured by t-test with one asterisk being p<0.05 and two asterisks being p<0.00 5 (n = 3).
(TIF)

Figure S7 Effects of VPA or β-catenin siRNA on levels of β-catenin and E-cadherin in HaCaT keratinocytes. HaCaT cells were transfected with 100 nM β-catenin siRNA before VPA treatment. (A, B) Immunocytochemical analysis of β-catenin (A) or E-cadherin (B) (original magnification ×635). (C) Western blot analysis of β-catenin or E-cadherin.
(TIF)

Figure S8 Effects of β-catenin siRNA on VPA-induced HaCaT keratinocyte migration. HaCaT cells were transfected with 100 nM β-catenin siRNA before VPA treatment. Migrating cells were stained with crystal violet (original magnification ×40).
(TIF)

Figure S9 Effects of VPA, U0126, or LY294002 on activities of ERK and Akt in HaCaT keratinocytes. 10 µM U0126 or LY294002 was pre-treated for 1 h before VPA treatment. (A, B) Immunocytochemical analysis of p-ERK (A) or p-Akt (B) (original magnification ×400). (C) Western blot analysis of p-ERK or p-Akt.
(TIF)

Figure S10 Effects of U0126 or LY294002 on VPA-induced HaCaT keratinocyte migration. 10 µM U0126 or LY294002 was pre-treated for 1 h before VPA treatment. Migrating cells were stained with crystal violet (original magnification ×40).
(TIF)
