# Supplementary figures and images for: Valproic Acid Induces Cutaneous Wound Healing In Vivo and Enhances Keratinocyte Motility
Source: PLoS One. 2012 Nov 7;7(11):e48791. doi: 10.1371/journal.pone.0048791 (PMC3492241; doi:10.1371/journal.pone.0048791)

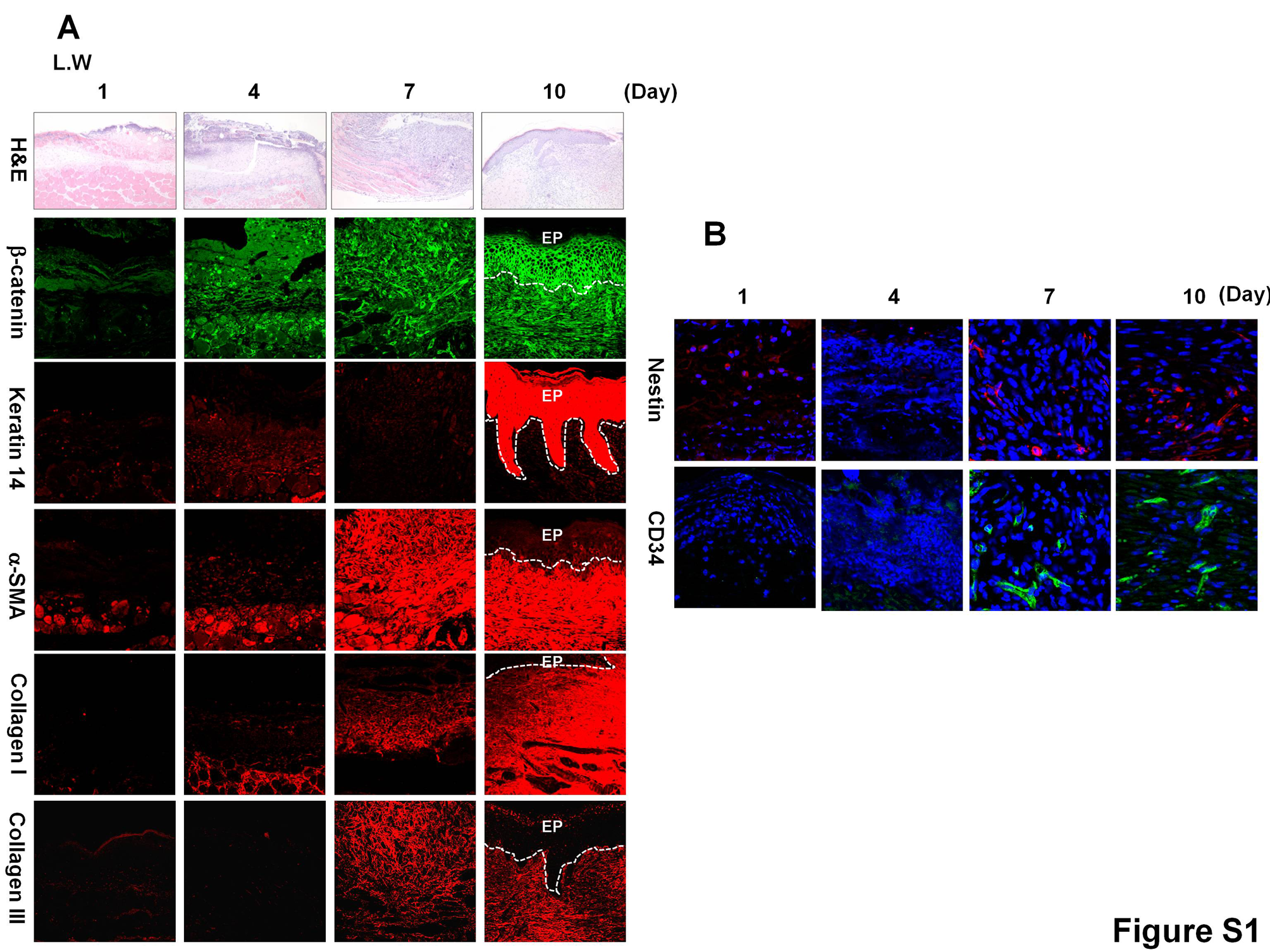

Supplement: Figure S1 — β-catenin status, wound healing and stem cell markers during the healing process in large wounds. Full-thickness wounds (diameter = 1.5 cm) were generated on the backs of 8-week-old C3H mice. Wounded tissues were excised from CH3 mice at 1, 4, 7, and 10 d post-wounding, and subjected to H&E staining and immunohistochemical analyses. (A) H&E staining (first row panels) (original magnification ×100) and immunohistochemical staining for β-catenin, keratin 14, α-SMA, collagen I, and collagen III (other row panels) in the wounds (original magnification ×200). EP, epidermis. (B) Immunohistochemical analysis of Nestin or CD34 in the wounds at 1, 4, 7, and 10 d post-wounding (original magnification ×635). (TIF) [file pone.0048791.s001.tif]

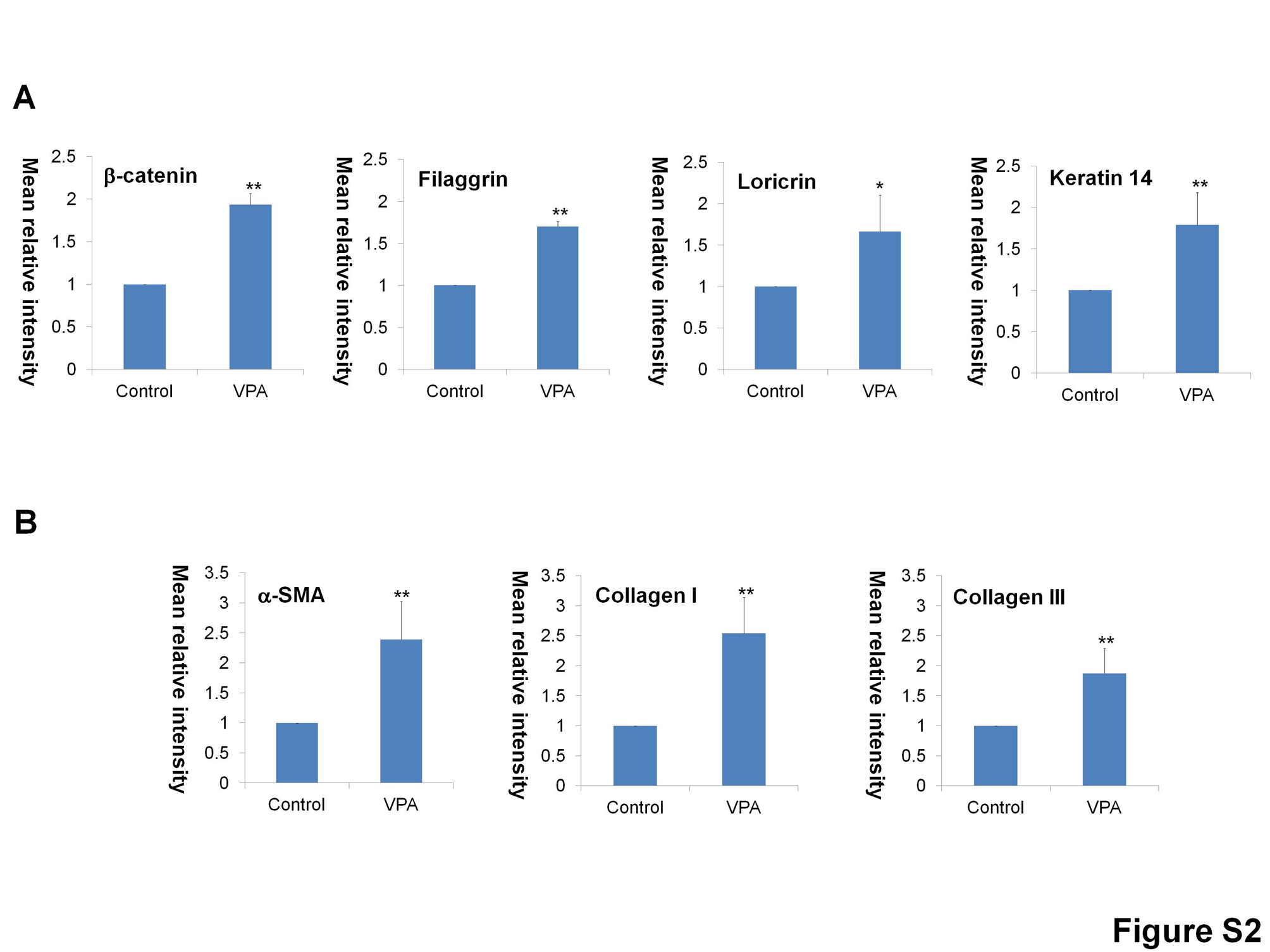

Supplement: Figure S2 — Effects of VPA on levels of β-catenin, wound healing markers in small wounds. The wounded skin of 8-week-old male C3H mice was treated daily with 500 mM VPA for 7 days. Wounded skin was fixed in paraformaldehyde overnight. (A) Immunohistochemistry was performed with β-catenin, filaggrin, loricrin, or keratin 14 antibodies in the neo-epidermis of control and VPA-treated wounds. The protein levels were quantified using TissueQuest analysis software. Asterisks denote the significant differences between control and test groups as measured by t-test with one asterisk being p<0.05, two asterisks being p<0.005 (n = 5). (TIF) [file pone.0048791.s002.tif]

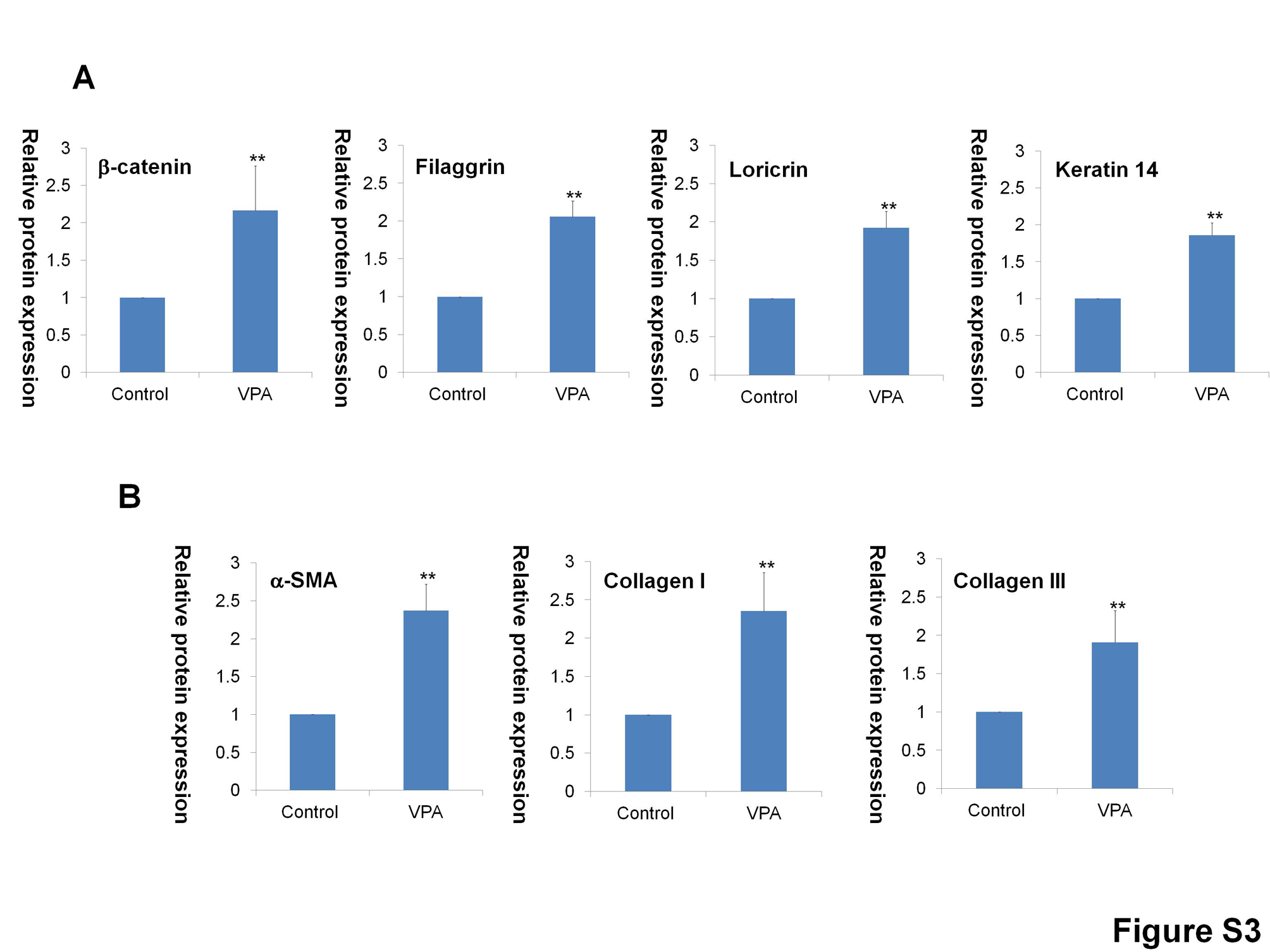

Supplement: Figure S3 — Effects of VPA on levels of β-catenin, wound healing markers in small wounds. The relative protein expression was calculated as the ratio of each protein level to α-tubulin level. The software used for the quantification was Multi-Gauge V 3.0 (Fujifilm). Asterisks denote the significant differences between control and test groups as measured by t-test with two asterisks being p<0.005 (n = 5). (TIF) [file pone.0048791.s003.tif]

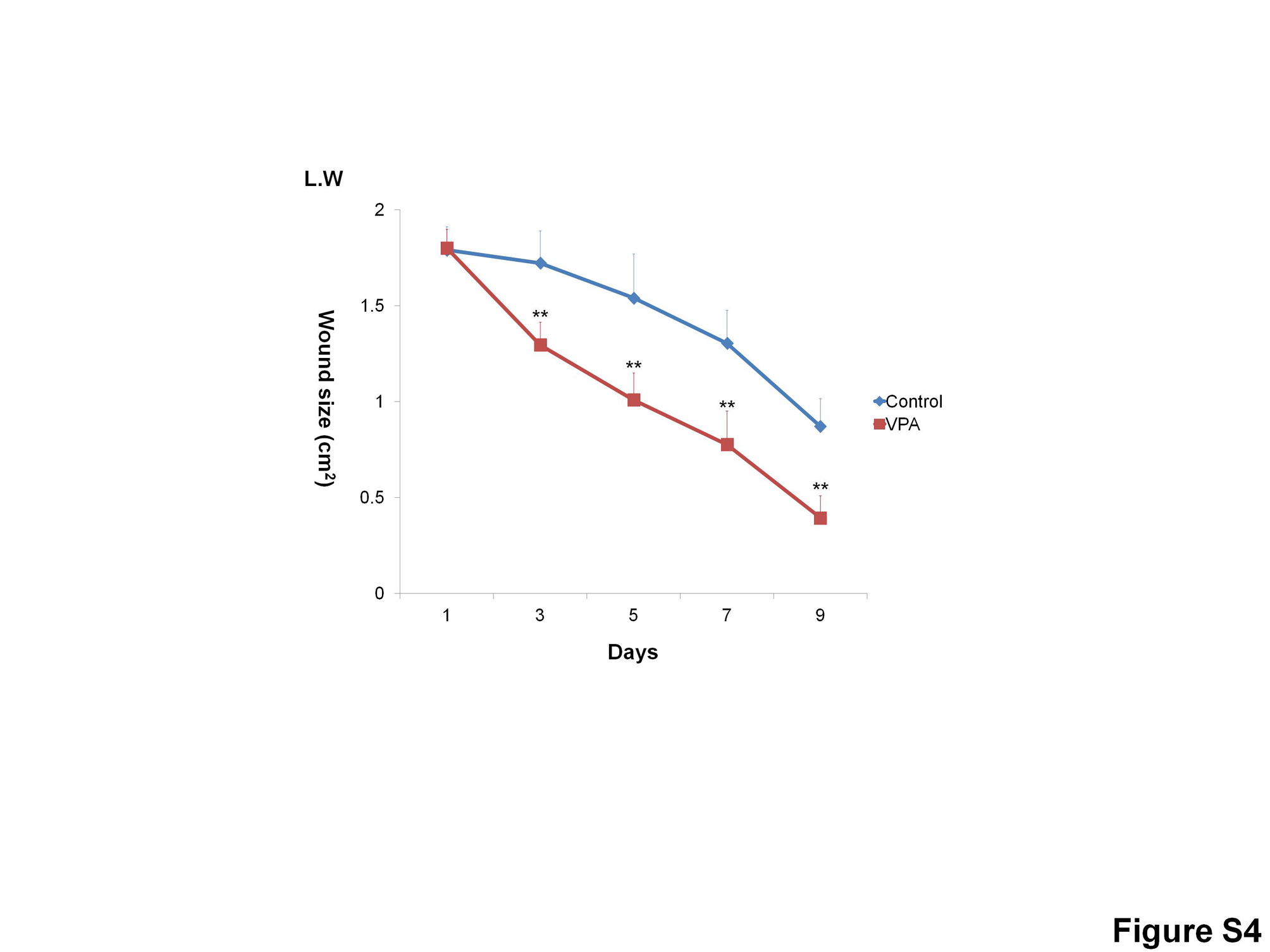

Supplement: Figure S4 — Quantitative data for effects of VPA on cutaneous wound healing in large wounds. A full-thickness skin excision (diameter = 1.5 cm) was made on the backs of 8-week-old C3H mice, and 500 mM VPA was topically applied to the wounds daily (Figure 4A). Wound sizes were measured at 1, 3, 5, 7, and 9 d after wounding. Asterisks denote the significant differences between control and test groups as measured by t-test with two asterisks being p<0.005 (n = 10). (TIF) [file pone.0048791.s004.tif]

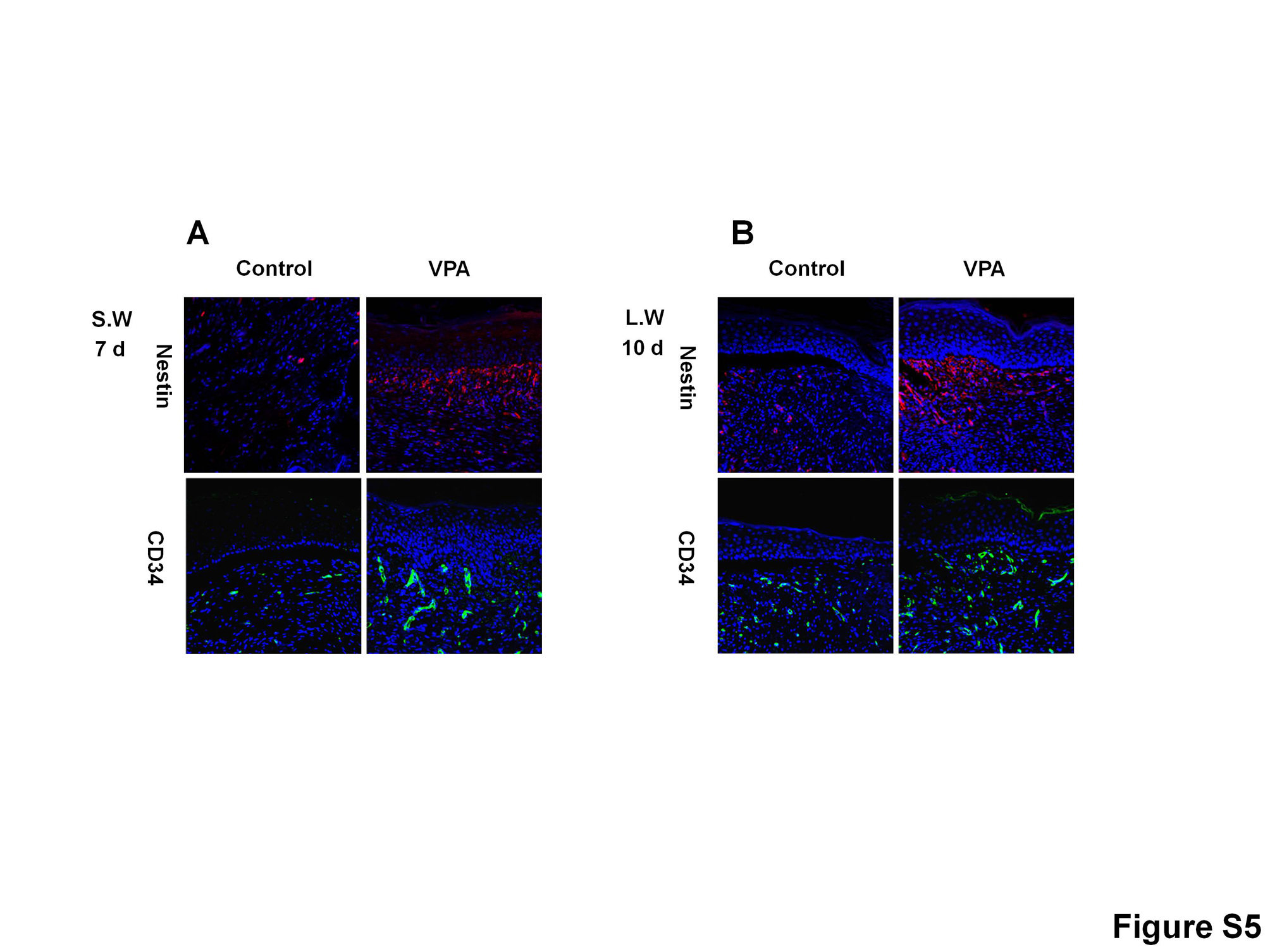

Supplement: Figure S5 — Effects of VPA on the expression of stem cell markers in wounds (Low magnification images). A full-thickness skin excision (diameter = 0.5 cm or 1.5 cm) was made on the backs of 8-week-old C3H mice, and 500 mM VPA was topically applied to the wounds daily. (A) Lowly magnified image of Fig. 5A (original magnification ×200). (B) Lowly magnified image of Fig. 5B (original magnification ×200). (TIF) [file pone.0048791.s005.tif]

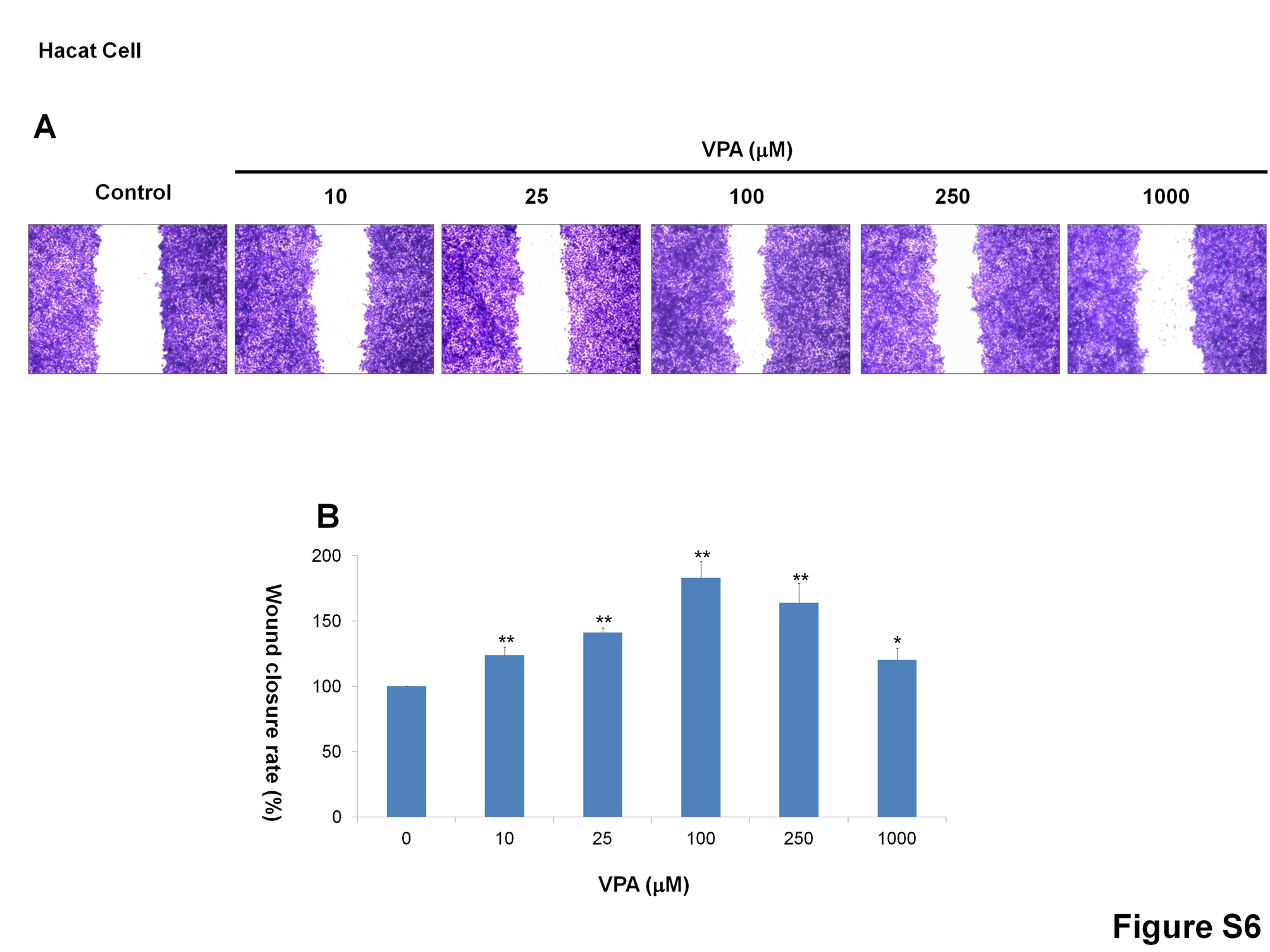

Supplement: Figure S6 — Effects of VPA concentration on HaCaT HaCaT keratinocyte migration. Hacat cells were treated with different concentrations of VPA for 24 hours. (A) Migrating cells were stained with crystal violet (original magnification ×40). (B) The relative wound closure rate was measured using NIS-Elements imaging software. Asterisks denote the significant differences between control and test groups as measured by t-test with one asterisk being p<0.05 and two asterisks being p<0.00 5 (n = 3). (TIF) [file pone.0048791.s006.tif]

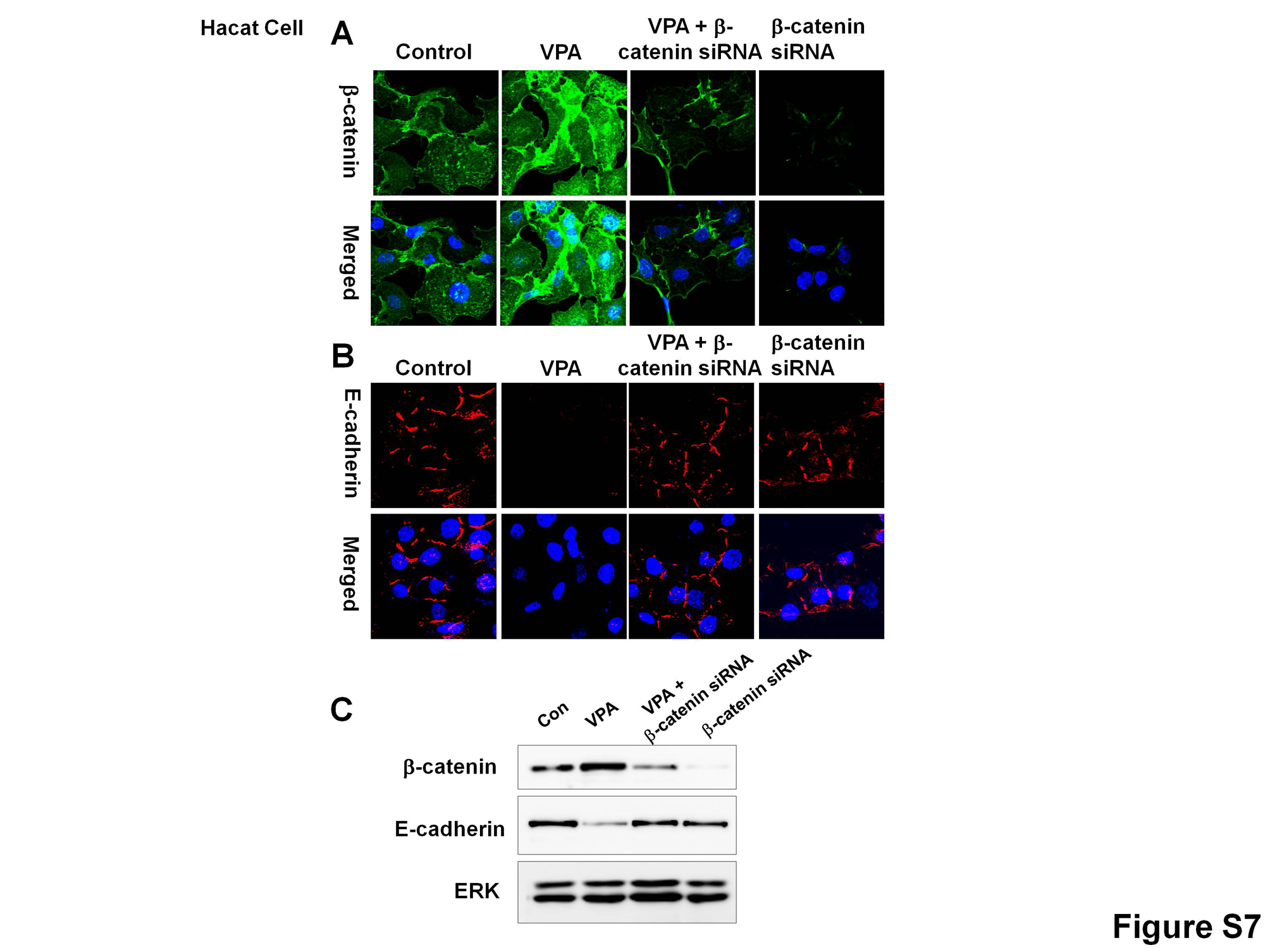

Supplement: Figure S7 — Effects of VPA or β-catenin siRNA on levels of β-catenin and E-cadherin in HaCaT keratinocytes. HaCaT cells were transfected with 100 nM β-catenin siRNA before VPA treatment. (A, B) Immunocytochemical analysis of β-catenin (A) or E-cadherin (B) (original magnification ×635). (C) Western blot analysis of β-catenin or E-cadherin. (TIF) [file pone.0048791.s007.tif]

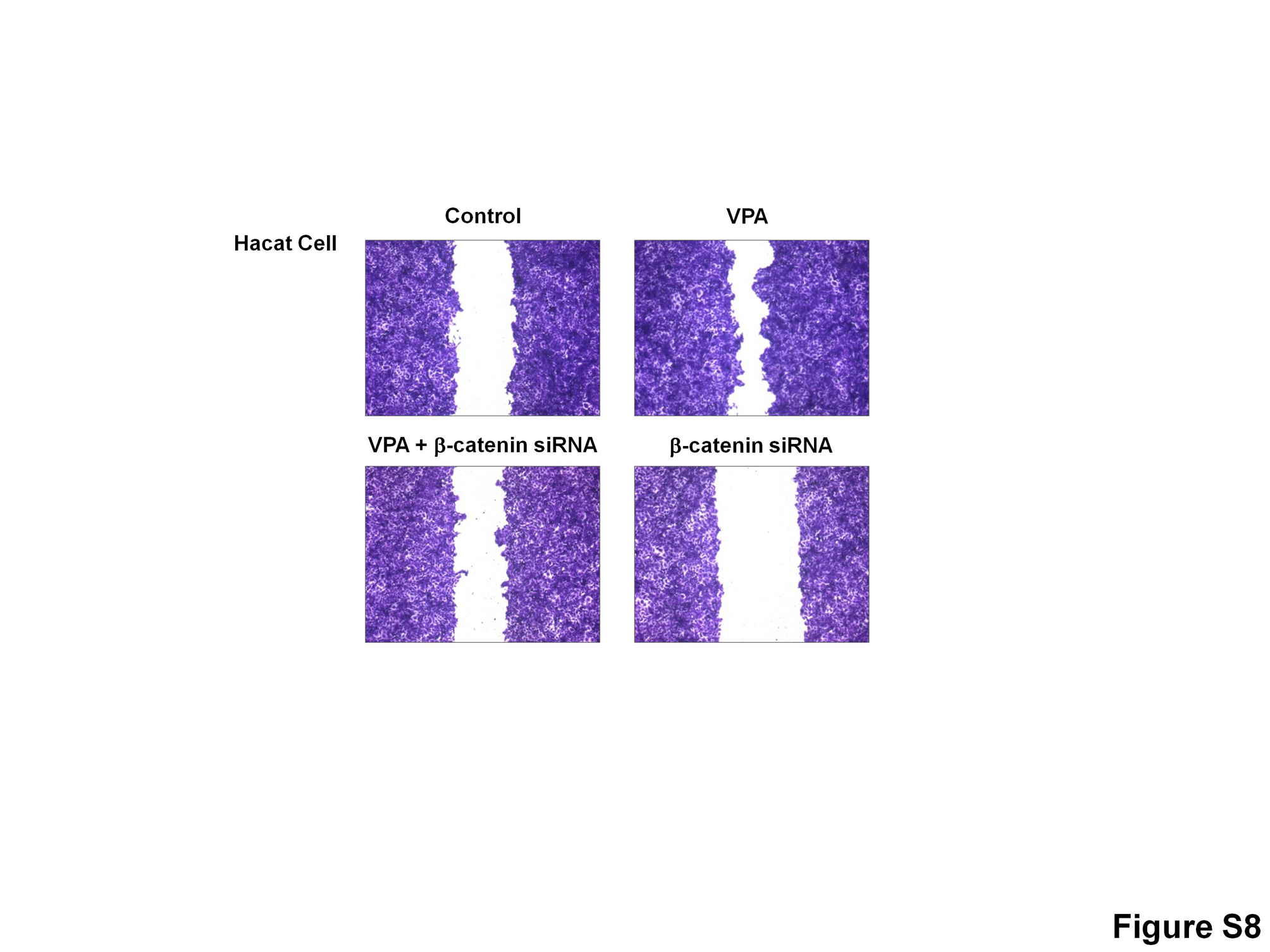

Supplement: Figure S8 — Effects of β-catenin siRNA on VPA-induced HaCaT keratinocyte migration. HaCaT cells were transfected with 100 nM β-catenin siRNA before VPA treatment. Migrating cells were stained with crystal violet (original magnification ×40). (TIF) [file pone.0048791.s008.tif]

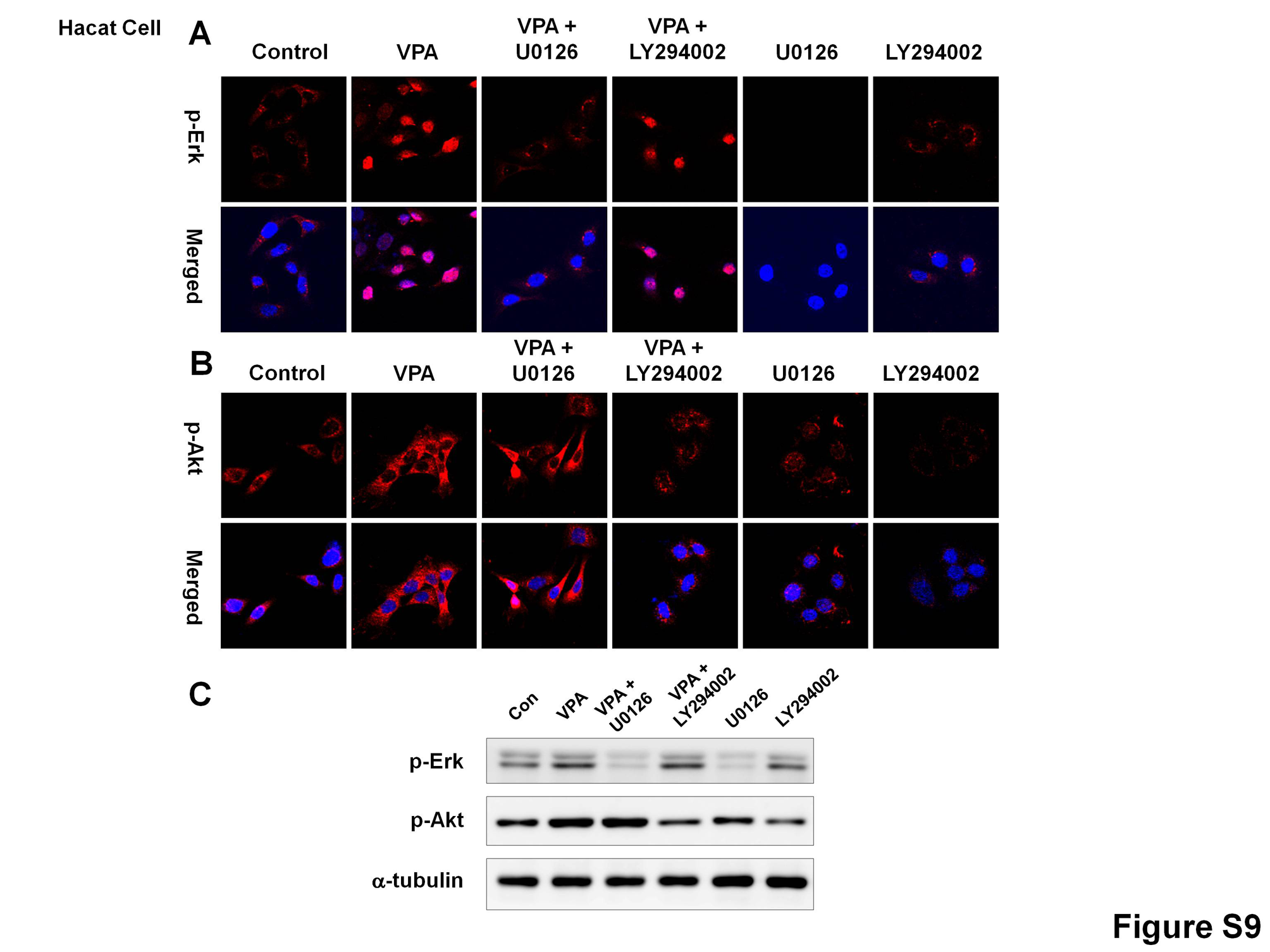

Supplement: Figure S9 — Effects of VPA, U0126, or LY294002 on activities of ERK and Akt in HaCaT keratinocytes. 10 µM U0126 or LY294002 was pre-treated for 1 h before VPA treatment. (A, B) Immunocytochemical analysis of p-ERK (A) or p-Akt (B) (original magnification ×400). (C) Western blot analysis of p-ERK or p-Akt. (TIF) [file pone.0048791.s009.tif]

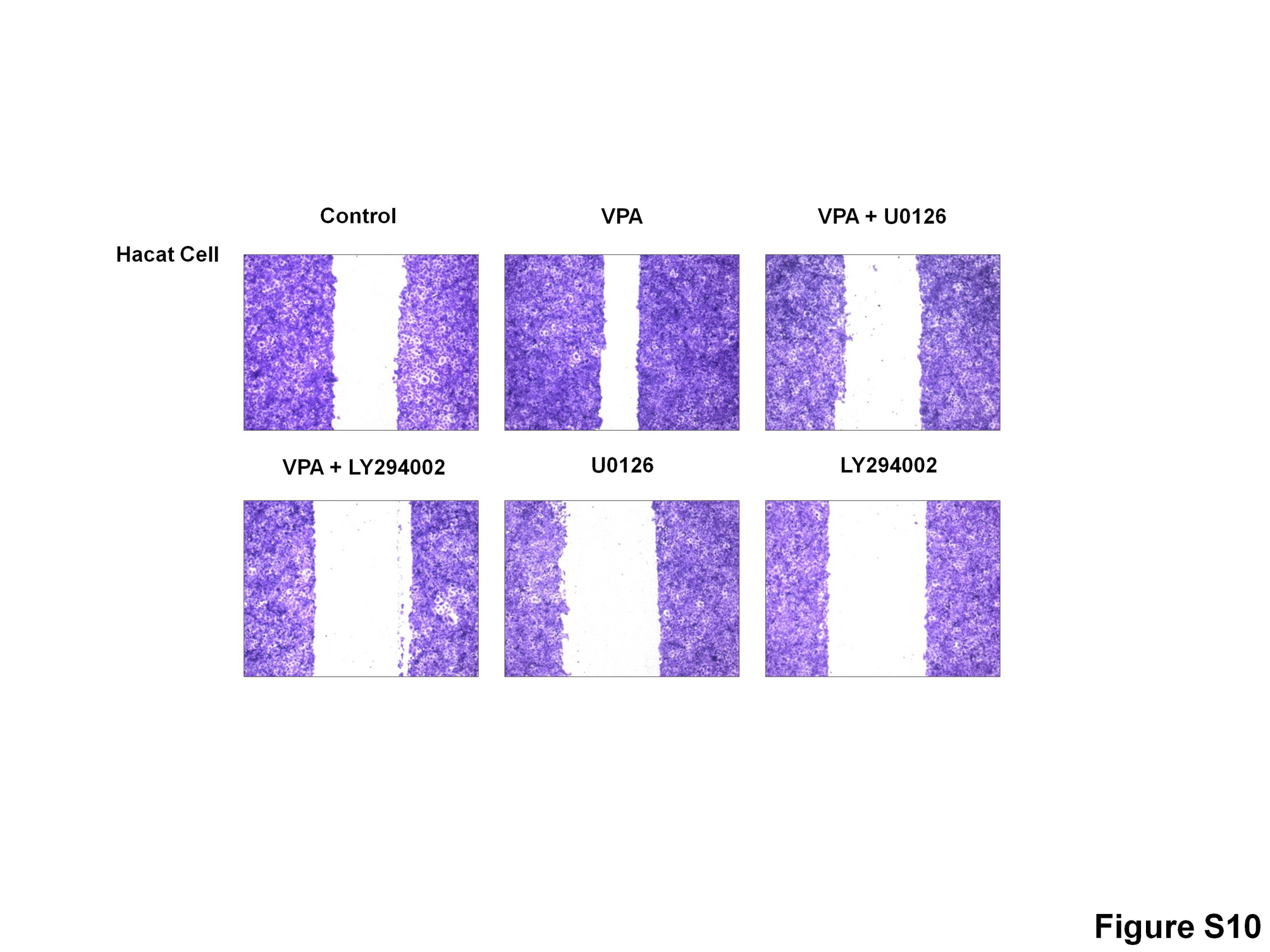

Supplement: Figure S10 — Effects of U0126 or LY294002 on VPA-induced HaCaT keratinocyte migration. 10 µM U0126 or LY294002 was pre-treated for 1 h before VPA treatment. Migrating cells were stained with crystal violet (original magnification ×40). (TIF) [file pone.0048791.s010.tif]
